# Substitution and Adulteration

**Published:** 1900-03

**Authors:** 

**Affiliations:** New York


### SUBSTITUTION AND ADULTERATION.

*To the Editor:*

*Sir.*—We take the liberty of sending separately a booklet, which we commend to your careful consideration. Owing to the extensive demand for our preparations they are being largely counterfeited, substituted and adulterated, and this is not only an injustice to ourselves but an outrage upon the physician who relies upon the purity of the drugs prescribed. We would esteem it a great favor if you would comment upon this matter and caution physicians against counterfeit phenacetin, aristol, sulfonal, trional, and others of our preparations. All the products sold by us bear on the label the name of the

"FARBENFABRIKEN OF ELBERFELD CO., 40 STONE STREET, NEW YORK."

We know that the majority of American physicians are making use of our preparations in their daily practice, and we shall be glad to avail ourselves of your kind offices by urging upon them the necessity of personally informing themselves whether the drug dispensed by their pharmacists is the genuine or some substituted or adulterated article. By doing so you will not only remedy a wrong committed against us, but also confer a great service upon the medical profession, which is so vitally interested in the purity of medicaments.

Yours very truly,
FARBENFABRIKEN OF ELBERFELD CO.

NEW YORK, January 31, 1900.

---

THE Pasteur Institute has declined a request from the Boers to send them a supply of antiplague serum, giving as a reason for this refusal that there was not enough serum in Paris to control an epidemic of the plague should it gain a footing in the city.—*Medical Age.*

